# Supplementary material for: Optically addressable spin defects coupled to bound states in the continuum metasurfaces
Source: Nat Commun. 2024 Mar 5;15:2008. doi: 10.1038/s41467-024-46272-1 (PMC10914779; doi:10.1038/s41467-024-46272-1)
Supplement: Supplementary file 1 — Supplementary Information file [file 41467_2024_46272_MOESM1_ESM.pdf]

Supplementary information for: Optically addressable spin defects coupled to bound states in the continuum metasurfaces

L. Sortino et al.

Supplementary Note 1: hBN metasurfaces Q factors

Supplementary Note 2: Transmission of hBN qBIC metasurfaces

Supplementary Note 3: PL enhancement of coupled spin defects

Supplementary Note 4: ODMR Signal-to-Noise ratio analysis

Supplementary Note 5: qBIC electric field numerical simulations

Supplementary Note 6: Radiation patterns of in-plane and out-of-plane dipoles coupled with hBN metasurfaces.

Supplementary Note 1: hBN metasurfaces Q factors

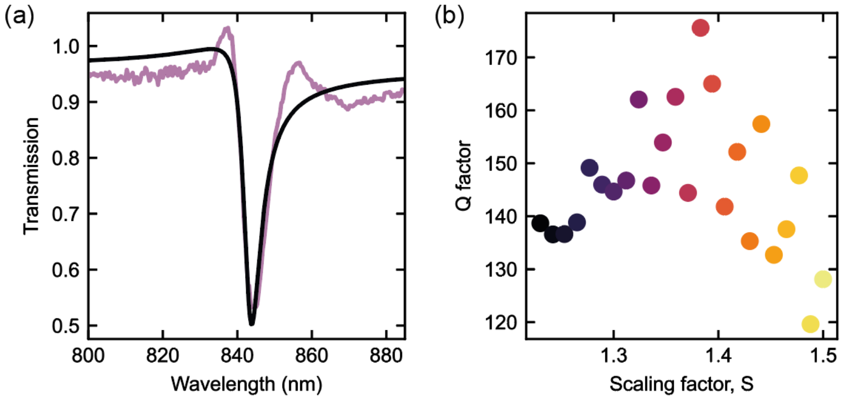

Supplementary Figure 1: (a) Transmission spectrum (in purple) of a fabricated hBN metasurface fitted with a Fano function (in black). (b) Extracted Q factors values for metasurfaces with different scaling factors.

Supplementary Note 2: Transmission of hBN qBIC metasurfaces

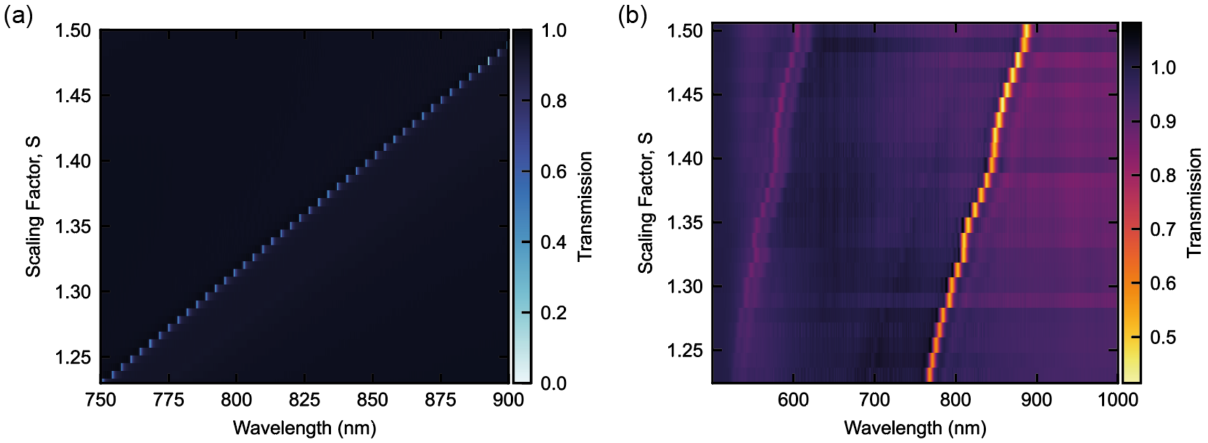

Supplementary Figure 2: (a) Numerically FDTD calculated transmission spectra of hBN qBIC metasurfaces on glass substrate with (ΔL = 50 nm). (b) Transmission spectra of the fabricated hBN metasurfaces under parallel excitation. We observe the appearance of a second peak at higher wavelength. The fabricated samples exhibit a smaller Q factor than numerical simulations, owing to defects and imperfections introduced during the fabrication and ion irradiation processes.

Supplementary Note 3: PL enhancement of coupled spin defects

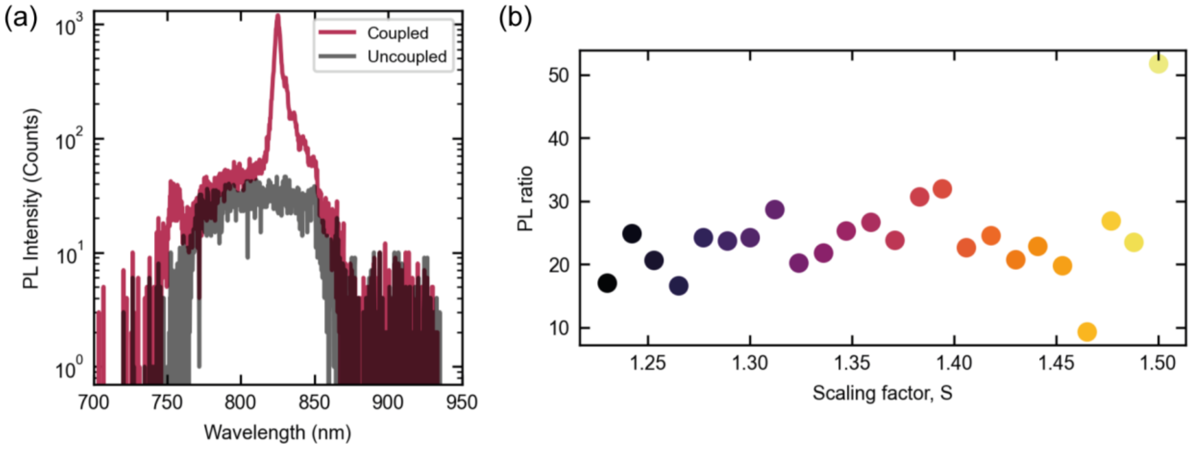

Supplementary Figure 3: (a) Raw PL emission spectra from V_B^- defects coupled to a qBIC metasurface (red) and on the unpatterned reference area (gray). (b) PL enhancement for defects coupled to hBN metasurfaces. The PL enhancement (I/I_0) is defined as the ratio between the integrated PL intensity of coupled defects (I) and the reference hBN PL emission (I_0), extracted from the data shown in Figure 3d in the main text.

Supplementary Note 4: ODMR Signal-to-Noise ratio analysis

The ODMR contrast for the unpatterned hBN reference sample is shown in Figure S4a, the data is integrated over 150 cycles with 1 ms integration time for each MW frequency. To compare with the patterned metasurface (Figure S4b), we define the Signal-to-Noise ratio ($S/N = P_{avg}/P_{std}$) as the ratio between the average value of the PL intensity (P_{avg}, solid line in Figure S4a,b) and the standard deviation of the PL signal at each MW frequency (P_{std}). We show the extracted value of this S/N ratio in Figure S4c. For unfiltered collection, we observe a small increase in the S/N ratio for the hBN metasurfaces from 21.3 to a value of 30.6. However, when inserting the 50 nm bandpass filter, we do not resolve a clear ODMR contrast anymore for the unstructured sample (Figure S4d). In contrast, the narrow emission from the metasurfaces funnels the light into the filter bandwidth and shows a clear ODMR contrast (Figure S4e). In Figure S4f, we plot the S/N ratio for the filtered collection and with 150 ms integration time, showing a large increase compared to the unfiltered case. The S/N is enhanced from approximately 1.42 times in the unfiltered case, up to 4.13 in case of bandpass filter, as shown in Figures S4c,f, where we plot the S/N ratio for whole MW range investigated. The dashed black line represents the average S/N value.

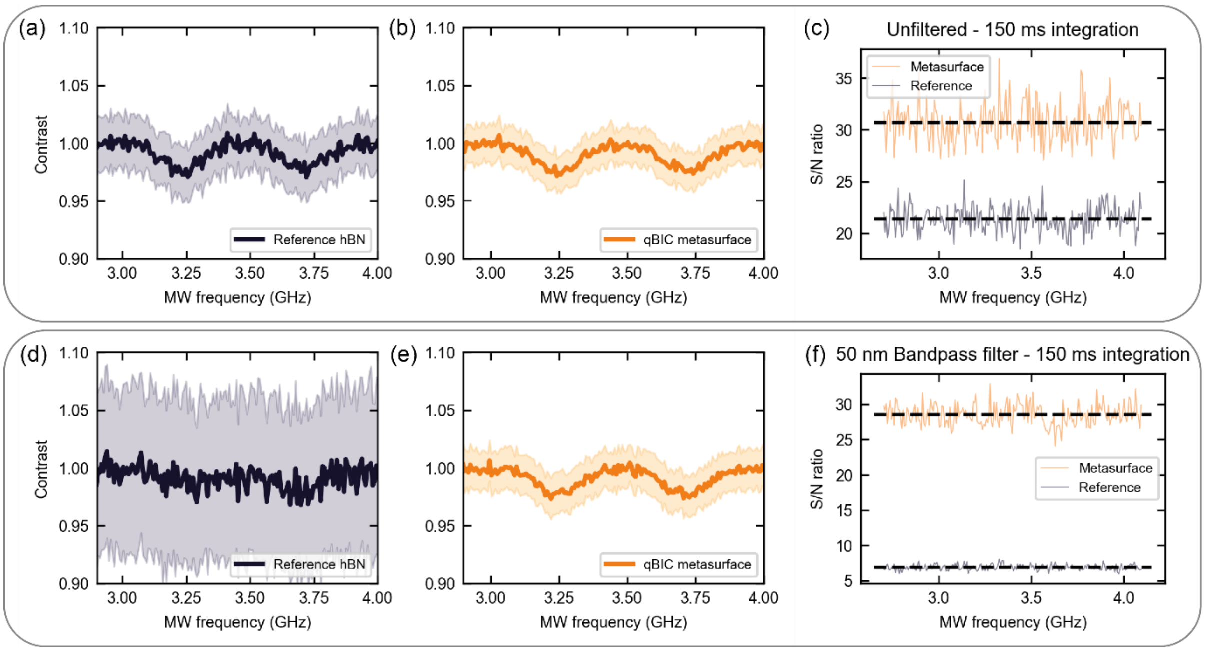

Supplementary Figure 4: (a-b) Unfiltered ODMR signal from the unstructured hBN reference sample (a) and a qBIC hBN metasurface (b). (c) Signal-to-Noise (S/N) ratio for unfiltered PL collection for the qBIC metasurface (orange) and unstructured sample (grey), averaged over 150 ms integration for each MW frequency. The dashed black line represents the average value. (d-e) Reproduction of Figure 3f-g from the main text of the manuscript: ODMR signal after a 775±25 nm bandpass filter is placed in the collection path, from the unstructured hBN reference sample (d) and a qBIC hBN metasurface (e), integrated over 150 ms for each MW frequency. (f) Signal-to-Noise ratio for Figures S4d-e. The dashed black line resents the average value.

Supplementary Note 5: qBIC electric field numerical simulations

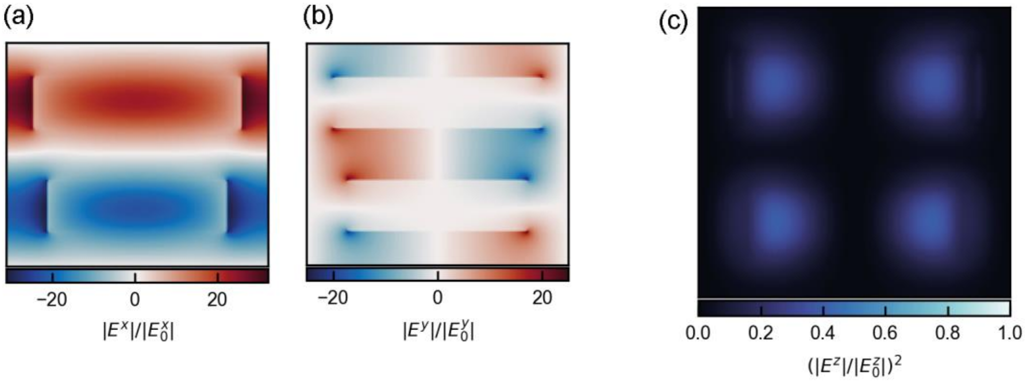

Supplementary Figure 5: (a-b) Profiles for the x (a) and y (b) electric field component, acquired at the qBIC resonance of an hBN metasurface, on SiO_2 substrates, with height of 160 nm and asymmetry of ΔL = 50 nm. (c) Out-of-plane electric field intensity for the same unit cell.

Supplementary Note 6: Radiation patterns of in-plane and out-of-plane dipoles coupled with hBN metasurfaces.

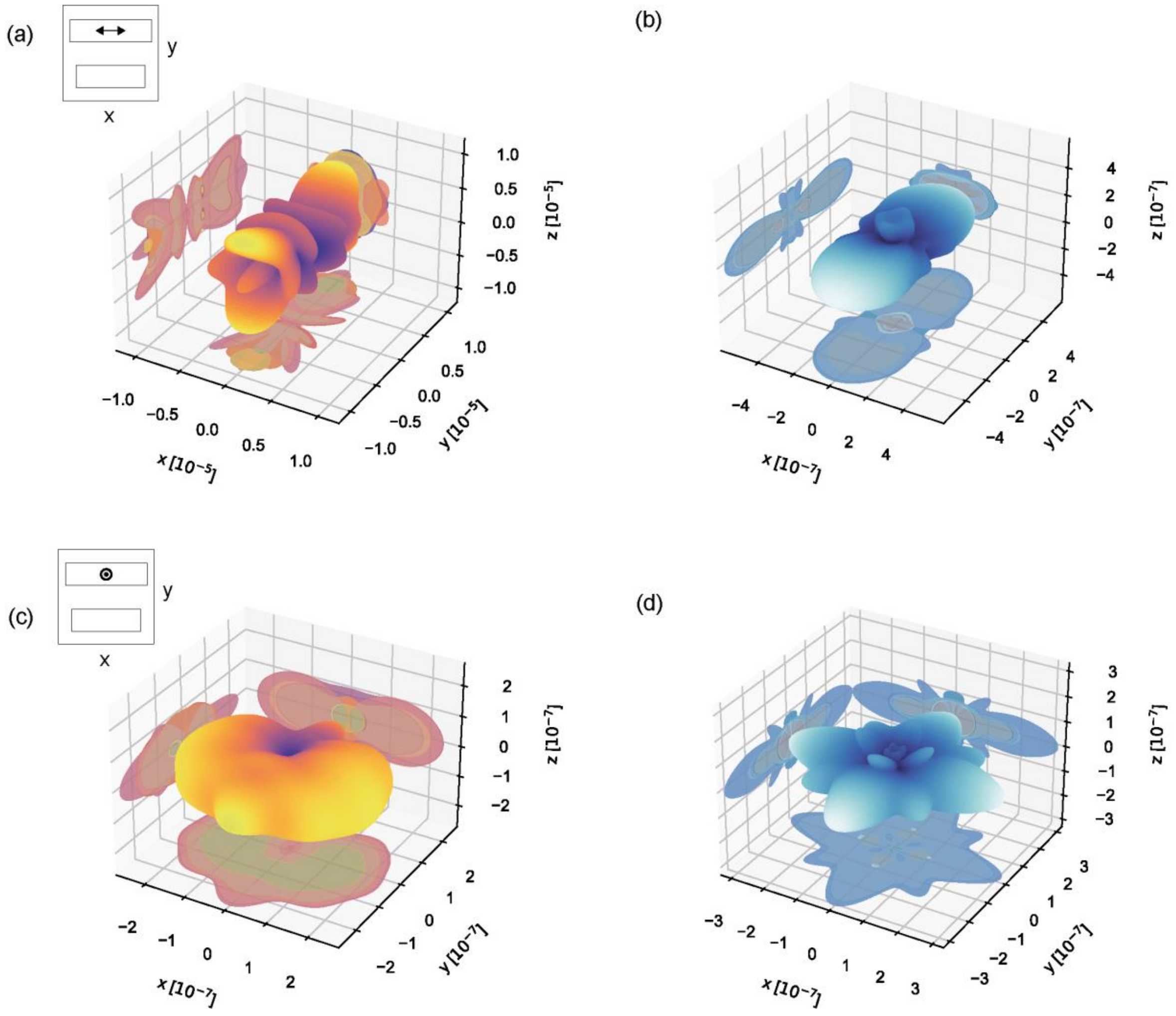

Supplementary Figure 6: (a-b) Numerical simulations of the far field patterns of the radiated electric field (E) for an in-plane dipole, aligned to the x-axis, coupled to the qBIC resonance (a) and in an hBN slab (b) of the same thickness. Inset: Position of the dipole considered for the numerical simulations, placed at the centre of the top nanorod, and with z position at half of the resonator height. (c-d) Numerically simulated far field patterns for an out-of-plane dipole emitter. For all the panels, the electric field component is projected along the relative axis.
